# Supplementary material for: Study of distribution and morphological characteristics of the trabecular bone in the uncinate process of the cervical spine using micro-computed tomography
Source: PLoS One. 2025 Jan 3;20(1):e0315640. doi: 10.1371/journal.pone.0315640 (PMC11698431; doi:10.1371/journal.pone.0315640)
Supplement: Attachment — Submitted filename: Micro-CT in the uncinate process_reviewer.docx [file pone.0315640.s002.docx]

**Application of Micro-computed Tomography in the Microstructure of Cervical Uncinate Processes Trabecular Bone**

**Running title**: Micro-CT in cervical uncinate processes

**Yunteng Hao^1+^,Yuan Ma^2+^, Shaojie Zhang^2^, Chaoqun Wang****^3^, Wei Wang^4^, Xiaohe Li^2^, Shang Gao^2^, Kun Li^2^, Jie Chen^2^, Haiyan Wang^2^, Yang Yang^1^, Mingjie Gao^2^, Jian Wang^2^, Zhijun Li^2^, Jun Shi^4*+^** **& Xing Wang^2*+^**

^1^Graduate School, Inner Mongolia Medical University, Hohhot, 010059, Inner Mongolia, China.

^2^Human Anatomy Teaching and Research Section (Digital Medical Center), Inner Mongolia Medical University Basic Medical College, Hohhot, 010059, China.

^3^Department of Imaging, Affiliated Hospital of Inner Mongolia Medical University, Hohhot, 010050, China.

^4^Department of Physiology, School of Basic Medicine, Inner Mongolia Medical University, Hohhot, Inner Mongolia 010059, China.

+These authors contributed equally to this work and share first authorship.

**^*+^**These authors contributed equally to this work and share corresponding authorship.

***Correspondence**

Xing Wang**^*+^**,

Email: wangxing197911@163.com

**Abstract**

This study aimed to observe the distribution and morphological characteristics of the trabecular bone in the uncinate process of the cervical spine on micro-computed tomography (Micro-CT) to identify the morphological characteristics of the microstructure in the uncinate process. Micro-CT scans were performed on 31 sets of C_3_-C_7_ vertebrae comprising 155 complete bone specimens. The measurement software of the Micro-CT system was used to identify the relevant parameters of the trabecular bone in the uncinate process. Trabecular bone in the uncinate process was mainly crisscrossed longitudinally and was continuous with the trabecular bone in the vertebral body. There was a significant difference in the bone surface area between the left and right sides; however, there were no other significant differences between the sides. Also, there was no obvious regularity in the change with the increase in vertebral sequence number. The peaks of each parameter were mostly concentrated in C_4_-C_6_, and there were significant differences between C_3_ and other cervical vertebrae for other parameters; differences between other vertebrae were not obvious. Micro-CT can be used to observe the arrangement of trabecular bone in the uncinate process of the cervical spine and allows the measurement of morphometric parameters. The trabecular bone in the uncinate process does not significantly differ between sides and vertebrae in adults.

**Keywords**: cervical spine; micro-computed tomography; osteoporosis; trabecular bone; uncinate process

**1 Introduction**

As an essential part of the cervical uncovertebral joint, the cervical uncinate process can lead to compression of peripheral nerves and blood vessels when it suffers from fracture, hyperplasia, and trauma, which can lead to cervical spine-related lesions such as vertebral artery-type and nerve root-type cervical spondylopathies1-3. Current research on the cervical uncinate process focuses on its external morphology4-6. However, the cervical uncinate process is crucial for maintaining the stability of the cervical spine, so there is an urgent need for an in-depth study of the microstructural parameters related to the cervical uncinate process.

The cervical uncinate process is the gradual formation as the development of the cervical vertebrae, spinal motion, the overall loading of the cervical spine, and other mechanical requirements increase with age. Bones exhibit macroscopic and microstructural variability in adapting to different mechanical environments in the body, suggesting a correlation between bone structure and stress7-9. A comparison of the parameters of the trabeculae in the pedicles, articular processes, vertebral plates, and spinous processes in the lower cervical spine revealed that microstructural differences also existed at different sites10. In another study, the articular processes were divided into four regions of interest, and the microstructures were found to differ between sides, vertebral sequences, and regions11. Yang et al. [12] divided the vertebral body into dorsal and ventral sides. They found that the volume, volume fraction, and connectivity of dorsal trabeculae were significantly larger than those of the ventral side. In contrast, the separation of dorsal trabeculae was significantly lower than that of the ventral side. Therefore, partitioning and exploring the same bone can allow the microstructural and mechanical changes within different regions of the same site to be observed and reveal its changing characteristics more comprehensively.

In this study, the cervical lordosis was scanned by Micro-CT, and the region of interest was selected. The microstructural parameters (BV/TV, Tb. N) were obtained for the comparative analysis between different vertebral sequences and different sides in order to reveal the differences between their microstructures and to explore the correlation between the microstructural parameters and the mechanical characteristics, to provide theoretical bases for the development of the cervical spine and diagnosis, treatment and prevention of cervical spondylosis caused by the cervical lordosis.

**2 Materials and Methods**

**2.1 Specimen selection**

The Human Anatomy Department of Inner Mongolia Medical University provided 31 adult C3-C7 vertebrae, totaling 155 pieces. Based on the research purpose, the specimens were collected from 10 June 2018 to 30 December 2022. All specimens were necropsy for daily teaching, stored in shaded condition at room temperature, with no breakage or missing, gender and age unknown, and no information about the recipient. The study was approved by the Biomedical Ethics Committee of Inner Mongolia Medical University (YKD2018031). The statement confirms that all methods were performed per relevant guidelines and regulations.

**2.2 Scanning method and parameters**

The study arranged the cervical vertebrae in the longitudinal direction according to the sequence of C3-C7 vertebrae. It used a Hiscan XM Micro-CT machine (Suzhou Hesfeld Information Technology Co., Ltd.) and its accompanying analysis and measurement software (Hiscan Analyzer). The fixed scanning parameters were as follows: layer thickness 0.05 mm, layer spacing 0.05 mm, single exposure time 50 ms, tube voltage 60 kV, current 134 uA, image matrix 2000*1600, scanning imaging field of view 10 cm*8 cm, pixel size of 0.05*0.05. Then, the study saved the finished images in the Dicom format on a Lenovo P320 workstation (Suzhou Haisfield Information Technology Co., Ltd.). The study selected the cervical uncinate process as the region of interest (ROI), used the built-in measurement software to extract the microstructure of the ROI, to observe the morphological features of the cervical uncinate process after three-dimensional reconstruction(Figures 1 and 2), and to measure the relevant parameters as shown in Table 1.

**2.3 Statistical methods**

We collected the raw data, recorded them in Excel tables, and imported them into IBM SPSS Statistics for Windows, version 25.0 (IBM Corp. Armonk, NY, USA) for statistical analysis. The data were expressed as mean ± standard deviation values($\bar{x}$±s). Comparisons between left and right sides were performed by paired-sample t-test; comparisons between different vertebral sequences were performed by one-way analysis of variance (ANOVA), and the LSD method was used to test for homogeneity of variance, while the Kruskal-Wallis test, which is a nonparametric test, was used to test for non-uniformity of variance. The test level *α*=0.05 was established, and *P*<0.05 was taken as a significant difference.

**3 Results**

**3.1 Comparison of morphometric parameters of the trabeculae bone in the cervical uncinate process between the left and right sides**

The left side BS was more significant than the right side (*P*<0.05) in all vertebrae except C3 (*P*>0.05). The Tb. Sp was significantly different only on the right and left sides of C6 (*P*<0.05), and there was no significant difference between the sides of the other vertebrae. The left sides did not differ from the right sides of any vertebrae in other parameters, including TV, BV, BS/BV, BV/TV, BS/TV, and Tb. Th, Tb. N, Eu. N, Conn, DA, and SMI(Figure 3).

**3.2 Comparison of various morphometric parameters of the trabecular bone in the cervical uncinate process between different vertebral sequences**

The TV was spiky, with the peak at C5, and there was no significant difference except between C3 and C4, C5 (P<0.05). The BV was generally wavy, but its mean value was trending more gently, with the peak at C5, only the C5 differed significantly from the C3, C4, and C7, and the rest were insignificant (P>0.05). The Tb.Th, Tb. Sp and Tb. N was flat, with slight differences between the values, and none differed between groups (P>0.05). The Eu. N was wavy, with the peak value located at C4, and the C3 differed significantly from the C6 and C4, and the Cs differed significantly from the C6 (P<0.05). The Conn was wavy, with the peak located at C6, and there were significant differences between C3 and Cs, between C4 and Cs, and C6 (P<0.05). The DA showed a sharp peak with increasing vertebral order, and the peak was located at C5, but there was no significant difference between the groups (P>0.05). The SMI showed a shallow ‘V’ shape, with the lowest value located at C4, the only significant difference was between C4 and C7 (P<0.05), and the other groups were insignificant(P>0.05). In summary, we found a pattern of change in the mean value of each index, where the peaks (valleys) were mainly located between C5/ C4-6, the common sites of cervical spondylosis (Figure 4).

**4 Discussion**

Trabeculae are extensions of the cortical bone within the cancellous bone (i.e., trabeculae are connected to the cortical bone),  in mostly spongy or honeycomb shape, aligned in the same direction as the pressure and tension exerted on the bone. They play a role in supporting and reducing weight, cushioning, and containing bone marrow, as well as responding and adapting to deformation13,14.  Bone trabeculae are widely distributed in the human body, constituting 80% of the bone surface, and contain various structures such as collagen, minerals, and bone tissue cells, which are the sites of various metabolic processes and an essential origin of the blood system. However, the internal microstructure of bone trabeculae cannot be observed by X-ray, CT, and MR in current imaging examinations. In contrast, the high resolution of Micro-CT can observe the pattern of trabecular arrangement and morphological characteristics of bone trabeculae in the cortical bone and accurately calculate the metrological and morphological parameters of the trabeculae in the ROI area by using relevant analysis software [15-18].  Currently, Micro-CT has been used in a large number of applications in the evaluation of bone microarchitecture and has become a routine test in the field of osteoporosis research through its high-resolution and clear observation of the pattern of bone trabecular arrangement and morphological characteristics within the bone cortex.

Micro-CT also obtains morphological parameters of bone trabeculae by 3D reconstruction and measurement, of which BV/TV, Tb.Th, Tb.N, Tb.Sp, and SMI are currently the most commonly used parameters. Scholars have investigated the specific significance of bone microstructural parameters in the material properties of bone trabeculae in animal or human samples[19-21]. They found that BV/TV is closely related to the apparent maximum stress, force load, elasticity, and stiffness of bone trabeculae, which is the primary indicator for evaluating the trabecular microstructure[22-24]. Yamada et al [25]tested the lumbar spine for compression damage and reported that BV/TV was strongly correlated with vertebral bone strength, but the correlation with Tb.Th was low. In this study, we found that the maximum value of BV/TV of the uncinate process was located at C_5_, indicating that the stress concentration point of the cervical uncinate process was at C_5_. Therefore, the C_5_ uncinate process hyperplasia was earlier and more prevalent, which is consistent with the favourable site of cervical spondylosis due to the uncinate process lesion.

Tb.Th, Tb. Sp and Tb. N constitutes the basic parameters of spatial morphology of bone trabeculae. We perform calculations using the spherical fitting method, where the fitting object is the bone trabeculae in calculating Tb. Th, whereas the Tb. Sp fitting object was the space between the bone trabeculae^26^, it can directly reflect the characteristics of bone trabecular changes [27]. In this study, we found that the basic morphological parameters of the bone trabeculae within the uncinate process did not differ significantly between the side aliases and between the vertebrae, suggesting that the morphological characteristics of the bone trabeculae within the uncinate process of each vertebra are stable.

Parameters such as Eu. N, Conn, and DA can indicate the state of bone trabeculae. Peng Jing et al. [28] studied the bone microstructure in the hip fracture region. They found that the interconnected structure of bone trabeculae allowed bone marrow, blood, and other components to reach all parts of the bone smoothly to meet the metabolic growth needs of the organism.

The mechanism of osteoporosis formation also includes a change in the shape of the bone trabeculae, i.e., from plate-like to rod-like[29]. SMI is a parameter that describes the proportion of plate and rod structures in the constituent structures of bone trabeculae. The SMI of bone trabeculae is 0 when plate-like and 3 when rod-like. When osteoporosis occurs, the SMI increases, the rod-like trabeculae increase, the plate-like trabeculae decrease, and the trabeculae become less compression-resistant. Lower SMI tended to occur in the skeleton's weight-bearing region, meaning there are more plate-like bone trabeculae[30]. The minimum value of SMI in this study was at C5, indicating a stress concentration point is in the C5 uncinate process, consistent with previous studies. The maximum value is located at C7, indicating that C7 has a poor loading capacity and is more susceptible to osteoporosis and fractures.

In order to adapt to the mechanical environment of the body, microstructural differences may occur between load-bearing and non-load-bearing bone, and blood plays an essential role in the bone remodeling process [31]. In this study, we compared and analyzed the pattern of change of bone trabecular parameters between the right and left aliases of the cervical uncinate process and found that the BS/BV of the left uncinate process was larger, considering that it might be related to the different sources of blood supply between the right and left sides of the uncinate process. The left subclavian artery supplying the left vertebral body and appendages (including the uncinate process) originates from the aortic arch. In contrast, the right subclavian artery supplying the right uncinate process originates from the brachiocephalic trunk, so the left side has a higher blood flow and better blood supply than the right side. In addition, the vertebral artery penetrates upward through the C1-6 transverse foramen. While the C7 transverse foramen is minor, within which there is only a tiny return of vertebral veins, and the blood supply to nourish the left uncinate process is relatively small, which may be one of the reasons for the most minor BS/TV and Tb. Th of the C7 uncinate process[32-34], and is also objectively determined that C7 is more prone to osteoporosis and degeneration.

**5 Conclusion**

In this study, we used Micro-CT to observe the microstructural characteristics of the internal bone trabeculae of the uncinate process and to summarise the changing law of the microstructure of the C3-C7 uncinate process, thus finding that the C7 uncinate process has poor loading capacity and stress, and has a higher risk of injury, and that the C5 uncinate process is the site of most significant cervical uncinate stress, which leads to its earliest occurrence of hyperplasia. This study can provide a sufficient experimental, theoretical basis for preventing and diagnosing cervical spondylosis and related diseases.  However, due to the limitations of the scanning coils, Micro-CT can only scan isolated small specimens or live rats under anesthesia and is unable to scan dynamic structures in large animals or humans. In addition, this study focused only on the metrological and morphological parameters of the cervical uncinate process trabeculae. And it did not compare the age and sex of donors or different parts of the same vertebrae, which will be the focus of our continued exploration.

**ACKNOWLEDGMENTS**

The authors thank Suzhou Heathfield Information Technology Co., Ltd. for providing the specimen scanning and data analysis computer used in this research.

**References**

1. Wang H, et al. Mechanistic advances in osteoporosis and anti-osteoporosis therapies. MedComm 11;4(3):e244. https//:doi.org/10.1002/mco2.244 (2023).
2. Osteoporosis and bone salt disease branch of the Chinese Medical Association. China Orthopedic Loose disease survey survey and “health bone” special action results released. Chinese Journal of Osteoporosis and Bone Mineral Diseases. **12**(4):317-318. https//:doi.org/10.3969/j.issn.1674-2591.2019.04.001 (2019)
3. Al-Hashimi L, et al. Trabecular Bone Score Significantly Influences Treatment Decisions in Secondary Osteoporosis. J Clin Med. 20;12(12):4147. https//:doi.org/10.3390/jcm12124147.(2023)
4. Bouxsein ML, et al. Guidelines for assessment of bone microstructure in rodents using micro-computed tomography. Journal of Bone & Mineral Research.**25**(7).

<https://doi.org/10.1002/jbmr.141> (2019)

5. Greenwood C, et al. The micro-architecture of human cancellous bone from fracture neck of femur patients in relation to the structural integrity and fracture toughness of the tissue . Bone Reports, 3(C): 67-75.https//:doi.org/10.1016/j.bonr.2015.10.001 (2015)
6. Poilliot, A.,et al. The quantification of 3D-trabecular architecture of the fourth cervical vertebra using CT osteoabsorptiometry and micro-CT. Journal of orthopaedic surgery and research, 18(1), 297. https://doi.org/10.1186/s13018-023-03760-2 (2023)
7. Viero, A. , et al. Utility of micro-CT for dating post-cranial fractures of known post-traumatic ages through 3D measurements of the trabecular inner morphology.Scientific reports,12(1), 10543. <https://doi.org/10.1038/s41598-022-14530-1> (2022)
8. Neldam CA, et al. Application of high resolution synchrotron micro-CT radiation in dental implant osseointegration. Journal of cranio-maxillo-facial surgery : official publication of the European Association for Cranio-Maxillo-Facial Surgery. **43**(5):682-687.https//:doi.org/10.1016/j.jcms.2015.03.012 (2015)
9. Chin, K. E., et al. Comparison of micro-CT post-processing methods for evaluating the trabecular bone volume fraction in a rat ACL-transection model.Journal of biomechanics,49(14), 3559–3563. https://doi.org/10.1016/j.jbiomech.2016.08.024 (2016).
10. D'Andrea, L., et al. Mechanical Properties of Robocast Glass Scaffolds Assessed through Micro-CT-Based Finite Element Models. Materials (Basel, Switzerland), 15(18), 6344. <https://doi.org/10.3390/ma15186344> (2022)
11. Liu, H., et al. Effect of stimulation sites on the performance of electromagnetic middle ear implant: A finite element analysis. Computers in biology and medicine, 124, 103918. <https://doi.org/10.1016/j.compbiomed.2020.103918> (2020).
12. Yu, T., et al. Effects of immediately static loading on osteointegration and osteogenesis around 3D-printed porous implant: A histological and biomechanical study. Materials science & engineering. C, Materials for biological applications, 108, 110406. <https://doi.org/10.1016/j.msec.2019.110406> (2020)
13. Datta, S., et al. Bioprinting of radiopaque constructs for tissue engineering and understanding degradation behavior by use of Micro-CT. Bioactive materials, 5(3), 569–576. <https://doi.org/10.1016/j.bioactmat.2020.04.015> (2020).
14. Zhang, et al. A comparison of bioreactors for culture of fetal mesenchymal stem cells for bone tissue engineering. Biomaterials, 31(33), 8684–8695. <https://doi.org/10.1016/j.biomaterials.2010.07.097> (2010)
15. Hsiao, C. Y., et al. Calcitonin Induces Bone Formation by Increasing Expression of Wnt10b in Osteoclasts in Ovariectomy-Induced Osteoporotic Rats. Frontiers in endocrinology, 11, 613. <https://doi.org/10.3389/fendo.2020.00613> (2020)
16. Rodt, T., et al. Lung tumour growth kinetics in SPC-c-Raf-1-BB transgenic mice assessed by longitudinal in-vivo micro-CT quantification. Journal of experimental & clinical cancer research : CR, 31(1), 15. <https://doi.org/10.1186/1756-9966-31-15> (2012).
17. Liu, H., et al. Anti-infection mechanism of a novel dental implant made of titanium-copper (TiCu) alloy and its mechanism associated with oral microbiology. Bioactive materials, 8, 381–395. <https://doi.org/10.1016/j.bioactmat.2021.05.053> (2021)
18. Udagawa, et al. Micro-CT observation of angiogenesis in bone regeneration. Clinical oral implants research, 24(7), 787–792. <https://doi.org/10.1111/j.1600-0501.2012.02458.x.> (2013)
19. Ginn-Hedman,et al. Diffusible contrast-enhanced micro-CT improves visualization of stented vessels. Cardiovascular pathology : the official journal of the Society for Cardiovascular Pathology, 60, 107428. https://doi.org/10.1016/j.carpath.2022.107428 (2022)
20. Lee, Y. K. et al. Micro-CT and Histomorphometric Study of Bone Regeneration Effect with Autogenous Tooth Biomaterial Enriched with Platelet-Rich Fibrin in an Animal Model. Scanning, 2021, 6656791. <https://doi.org/10.1155/2021/6656791> (2021)
21. Nikodem A. Correlations between structural and mechanical properties of human trabecular femur bone. Acta of bioengineering and biomechanics, 14(2), 37–46.(2012)
22. Goulet, R. W., et al. The relationship between the structural and orthogonal compressive properties of trabecular bone. Journal of biomechanics, 27(4), 375–389. <https://doi.org/10.1016/0021-9290(94)90014-0> (1994).
23. Ding, Ming. et al. 3-D microarchitectural properties and rod- and plate-like trabecular morphometric properties of femur head cancellous bones in patients with rheumatoid arthritis, osteoarthritis, and osteoporosis. Journal of Orthopaedic Translation. 28. 159-168. https://doi.org/10.1016/j.jot.2021.02.002. (2021)
24. Barrett, J. M., et al. Cervical spine joint loading with neck flexion. Ergonomics, 63(1), 101–108. <https://doi.org/10.1080/00140139.2019.1677944> (2020)
25. Rieger, R.,et al. Micro-finite-element method to assess elastic properties of trabecular bone at micro- and macroscopic level. Morphologie : bulletin de l'Association des anatomistes, 102(336), 12–20. <https://doi.org/10.1016/j.morpho.2017.07.175> (2018)
26. SABET FA, et al. Nonlinear micro-CT based FE modeling of trabecular bone-Sensitivity of apparent response to tissue constitutive law and bone volume fraction. Int J Numer Method Biomed Eng, 34(4):e2941. <https://doi.org/10.1002/cnm.2941> (2018)
27. YAMADA S, et al. Correlation between vertebral bone microstructure and estimated strength in elderly women: An ex vivo HR-pQCT study of cadaveric spine. Bone. 120:459-464. <https://doi.org/10.1016/j.bone.2018.12.005> (2019)
28. Viveen, J.,et al. Three-dimensional cortical and trabecular bone microstructure of the proximal ulna. Archives of orthopaedic and trauma surgery, 143(1), 213–223. <https://doi.org/10.1007/s00402-021-04023-7> (2023).
29. Hildebrand, T. et al. A new method for the model-independent assessment of thickness in three-dimensional images. Journal of Microscopy, 185: 67-75. <https://doi.org/10.1046/j.1365-2818.1997.1340694.x> (1997)
30. REINA N, et al. BMI-related microstructural changes in the tibial subchondral trabecular bone of patients with knee osteoarthritis. J Orthop Res. 5(8):1653-1660. <https://doi.org/10.1002/jor.23459> (2017)
31. GREENWOOD C, et al. The micro-architecture of human cancellous bone from fracture neck of femur patients in relation to the structural integrity and fracture toughness of the tissue. Bone Rep. 3:67-75. <https://doi.org/10.1016/j.bonr.2015.10.001> (2015)
32. LI S, et al. Trabecular Microstructure and Damage Affect Cement Leakage From the Basivertebral Foramen During Vertebral Augmentation. Spine. 42(16):939-948.https://doi.org/10.1097/BRS.0000000000002073 (2017)

**Table**

**Table 1.** Morphometric parameters

| **Parameter** | **Abbreviation** | **Significance** |
| --- | --- | --- |
| Total volume | TV | Volume of the region of interest (mm^3^) |
| Bone volume | BV | Trabecular bone volume in the region of interest (mm^3^) |
| Bone surface | BS | Trabecular bone surface area in the region of interest (mm^2^) |
| Bone surface / bone volume | BS /BV | Indicates the area size of bone tissue per unit volume (1/mm) |
| Bone volume / tissue volume | BV/TV | The ratio of bone volume to tissue volume |
| Bone surface / total volume | BS/TV | Also known as bone surface area density, it can indirectly reflect bone mass (1/mm) |
| Trabecular thickness | Tb.Th | The average thickness of trabecular bone (μm); when osteoporosis occurs, the Tb.Th value decreases |
| Trabecular separation | Tb.Sp | The average width of the medullary cavity between trabecular bones (μm); an increase in Tb.Sp indicates increased bone resorption and which can be a sign of osteoporosis |
| Trabecular number | Tb.N | The number of intersections between bone tissue and non-bone tissue within a given length (1/mm); the Tb.N value decreases when osteoporosis occurs |
| Euler number | Eu.N | A measure of trabecular bone connectivity; the greater the Euler number, the greater the degree of osteoporosis |
| Connectivity | Conn | The degree of interconnection between trabecular bone structures (1/mm); when the connectivity reduced, with the bone loss diseases may occur |
| Degree of anisotropy | DA | The average intercept length in the region of interest of the uncinate process is the ratio of the long diameter to the short diameter of the ROI; in the early stage of osteoporosis, the DA of the load-bearing trabecular bone usually increases, and as the osteoporosis worsens, the DA decreases |
| Structure model index | SMI | The ratio of plate-like and rod-shaped trabecular bones; when osteoporosis occurs, plate-shaped bone trabeculae are reduced, rod-shaped bone trabeculae increase, and the SMI value increases |

**FIGURE LEGENDS**

**Figure 1.** Selection and three-dimensional reconstruction of trabeculae in the region of interest of the uncinate process

**Figure 2.** Trabecular arrangement of cervical vertebrae under Micro-CT(leftt), three-dimensional reconstruction results(right)

**Figure 3.** Comparisons between the left and right sides for the morphometric parameters of the trabecular bone of the uncinate process of the cervical spine. SMI, structure model index; Tb.Th, trabecular thickness; Tb.Sp, trabecular separation; Tb.N, trabecular number; TV, total volume; BV, bone volume; BS, bone surface; Conn, connectivity; DA, degree of anisotropy; Eu.N, Euler number

**Figure 4.** The trend in the morphometric parameters of the hook vertebra trabecula according to the vertebral number. SMI, structure model index; Tb.Th, trabecular thickness;	Tb.Sp, trabecular separation; Tb.N, trabecular number; TV, total volume; BV, bone volume; BS, bone surface; Conn, connectivity; DA, degree of anisotropy; Eu.N, Euler number

**Figure 1.** Selection and three-dimensional reconstruction of trabeculae in the region of interest of the uncinate process

**Figure 2.** Trabecular arrangement of cervical vertebrae under Micro-CT(left), three-dimensional reconstruction results(right)

**Figure 3.** Comparisons between the left and right sides for the morphometric parameters of the trabecular bone of the uncinate process of the cervical spine. SMI, structure model index; Tb.Th, trabecular thickness; Tb.Sp, trabecular separation; Tb.N, trabecular number; TV, total volume; BV, bone volume; BS, bone surface; Conn, connectivity; DA, degree of anisotropy; Eu.N, Euler number

**Figure 4.** The trend in the morphometric parameters of the hook vertebra trabecula according to the vertebral number. SMI, structure model index; Tb.Th, trabecular thickness;	Tb.Sp, trabecular separation; Tb.N, trabecular number; TV, total volume; BV, bone volume; BS, bone surface; Conn, connectivity; DA, degree of anisotropy; Eu.N, Euler number
